# *Anaplasma phagocytophilum*–infected Ticks, Japan

**DOI:** 10.3201/eid1111.050407

**Published:** 2005-11

**Authors:** Norio Ohashi, Megumi Inayoshi, Kayoko Kitamura, Fumihiko Kawamori, Daizoh Kawaguchi, Yuusaku Nishimura, Hirotaka Naitou, Midori Hiroi, Toshiyuki Masuzawa

**Affiliations:** * University of Shizuoka, Shizuoka, Japan; † Center of Excellence Program in the 21st Century, Shizuoka, Japan; ‡ Shizuoka Institute of Environment and Hygiene, Shizuoka, Japan

**Keywords:** Anaplasma phagocytophilum, anaplasmosis, Ixodes, Japan, p44 protein, 16S ribosomal RNA, dispatch

## Abstract

We report *Anaplasma phagocytophilum* infection of *Ixodes persulcatus* and *I. ovatus* ticks in Japan. Unique *p44*/*msp2* paralogs (and/or 16S rRNA genes) were detected in tick tissues, salivary glands, and spleens of experimentally infected mice. These findings indicate the public health threat of anaplasmosis in Japan.

*Anaplasma phagocytophilum* (formerly known as the agent of human granulocytic ehrlichiosis), *Ehrlichia phagocytophila*, and *E. equi* (*1*) are tickborne human pathogens of veterinary importance. They cause an emerging infectious and febrile systemic illness now known as human granulocytic anaplasmosis. The first case of human infection by *A. phagocytophilum* was reported in 1994 (*2*). Since then, an increasing number of cases have been recognized in the United States. Severities of this disease range from asymptomatic seroconversion to death, and severe illness is frequently documented. In Europe, the first human cases of this disease were described in 1997 (*3*), and serologic and polymerase chain reaction (PCR) analyses suggest that *A. phagocytophilum* is distributed throughout Europe and in some parts of the Middle East and Asia (*4**–**6*).

In nature, *A. phagocytophilum* is believed to be maintained in a tick-rodent cycle. The known vectors for this agent are *Ixodes* ticks, i.e., *Ixodes scapularis* and *I. pacificus* in the United States, *I. ricinus* mostly in Europe, and *I. persulcatus* in Russia (*7*) and China (*5*). Exposure to *A. phagocytophilum*–infected tick bites is the most common route of human infection, except for perinatal transmission or contact with infected mammalian blood (*8**,**9*).

In Japan, several *Ixodes* species, such as *I. persulcautus*, *I. ovatus*, and *I. monospinosus*, are potential vectors for transmission of *Borrelia* spp., *Rickettsia* spp., or *Ehrlichia* spp (*10**–**12*). However, little information is available regarding the ecologic and epidemiologic features of clinical cases of infection with *A. phagocytophilum* in Japan. We report infection with *A. phagocytophilum* in *Ixodes* ticks in central Japan determined by molecular epidemiologic approaches.

## The Study

**Figure 1:**
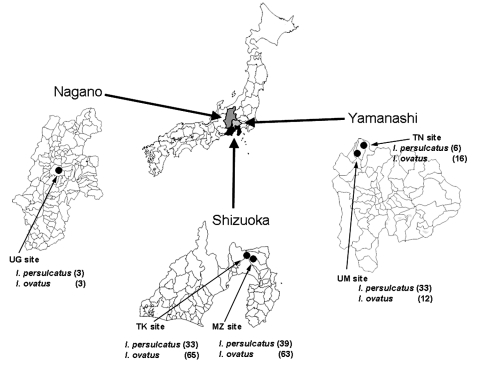
Areas in Shizuoka, Nagano, and Yamanashi Prefectures of Japan where Ixodes persulcatus and I. ovatus ticks were collected in 2003 and 2004. Closed circles indicate collection sites. Numbers of ticks collected at each site are shown in parentheses. UG, Utsukushigahara; TK, Takabachi; MZ, Mizugazuka; TN, Tennyosan; UM, Utsukushinomori.

In 2003 and 2004, a total of 273 unfed and adult *Ixodes* ticks (114 *I. persulcatus* and 159 *I. ovatus*) were collected in central Japan (Figure 1). Of these, 123 live ticks were dissected, and DNA was isolated from whole tissues of 73 ticks and salivary glands of 50 ticks by using the QIAamp DNA mini kit (Qiagen Inc., Valencia, CA, USA). For detection of *A. phagocytophlilum* DNA, a nested PCR using primers designed based on the highly conserved region of *p44*/*msp2* paralogs of (p3726 [5´-GCTAAGGAGTTAGCTTATGA-3´], p3761, p4183, and p4257) was conducted (12–14). Four (12.1%) of 33 *I. persulcatus* ticks collected at the Utsukushinomori (UM) site in Yamanashi Prefecture were positive by PCR (Table). Sixteen (7 *I. persulcatus* and 9 *I. ovatus*) (32%) of 50 salivary glands from ticks collected at the Takabachi and Mizugazuka sites in Shizuoka Prefecture were positive by PCR. Data indicated that *I. persulcatus* and *I. ovatus* in Japan are naturally infected with *A. phagocytophilum* and that ticks at certain sites are highly infected.

**Table:** Polymerase chain reaction (PCR) detection of Anaplasma phagocytophilum p44/msp2 paralogs from Ixodes ticks or spleens of mice experimentally infected with tick tissues

Collection site, year*	Whole tissue†		Salivary gland†	Experimental infection with ticks‡		Total
	Female	Male	Female	Female	Male	
*I. persulcatus*						
Yamanashi, 2004						
TN	0/2	0/4				0/6
UM	2/16	2/17				4/33
Nagano, 2004						
UG	0/3	0/0				0/3
Shizuoka, 2004						
TK			6/9			6/9
MZ			1/8			1/8
Shizuoka, 2003						
TK				0/14 (2)	0/10 (1)	0/24 (3)
MZ				0/22 (2)	0/9 (1)	0/31 (3)
Total	2/21	2/21	7/17	0/36 (4)	0/19 (2)	11/114 (6)
*I. ovatus*						
Yamanashi, 2004						
TN	0/8	0/8				0/16
UM	0/9	0/3				0/12
Nagano, 2004						
UG	0/1	0/2				0/3
Shizuoka, 2004						
TK			9/17			9/17
MZ			0/16			0/16
Shizuoka, 2003						
TK				0/32 (3)	1/16 (2)	1/48 (5)
MZ				0/26 (2)	0/21 (2)	0/47 (4)
Total	0/18	0/13	9/33	0/58 (5)	1/37 (4)	10/159 (9)

*TN, Tennyosan; UM, Utsukushinomori; UG, Utsukushigahara; TK, Takabachi; MZ, Mizugazuka. †No. positive/no. examined. One hundred twenty-three ticks were dissected (whole tissues from 73 ticks and salivary glands from 50) were individually examined by PCR. ‡No. of positive mouse spleens/no. of ticks examined (no. of mice used). Six to 15 ticks were pooled and homogenized (55 *I. persulcatus* and 95 *I. ovatus*), and intraperitoneally injected into ddY male mice.

We further examined the infection of immunocompromised mice with *A. phagocytophilum* in ticks by using the procedure described previously (*12*). Briefly, whole tissues from 150 live ticks (55 *I. persulcatus* and 95 *I. ovatus*) were pooled and intraperitoneally injected into 15 ddY male mice (6–15 pooled ticks per mouse) treated with the immunosuppressant cyclophosphamide. PCR was conducted with DNA isolated from blood and spleens of these mice. Only 1 of 9 spleens from *I. ovatus*-injected mice was positive by PCR (Table). We previously detected *Ehrlichia* spp. DNA in *I. ovatus*–injected mice, but did not detect *A. phagocytophilum* DNA in *I. ovatus*– or *I. persulcatus*–injected mice (*12*) because we used only a few immunocompromised mice, i.e., most had normal immune systems. Thus, we treated all 15 mice used in the present study with cyclophosphamide. Results indicate that *A. phagocytophilum* in *I. ovatus* can be infective for immunocompromised mice, although the efficiency of infection was low (1/95 [1.1%]).

**Figure 2:**
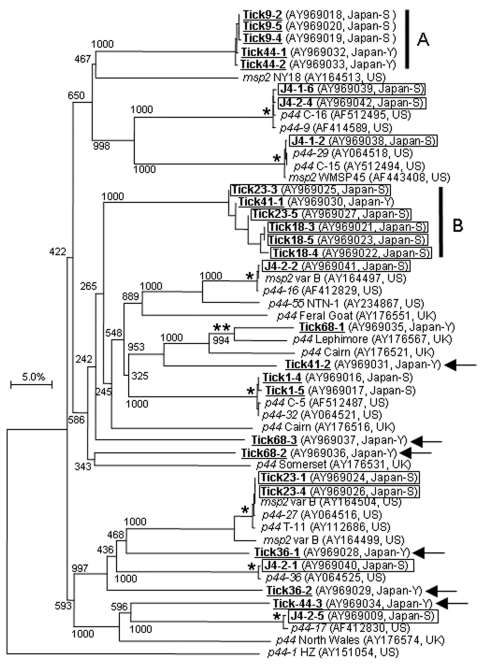
Phylogram of Anaplasma phagocytophilum p44/msp2 including Japanese paralogs. A) Cluster from Ixodes persulcatus. B) Cluster from I. ovatus, except for Tick41-1. The tree was constructed using the neighbor-joining method. Numbers on the tree indicate bootstrap values for branch points. Japanese p44/msp2 paralogs from I. persulcatus and I. ovatus are underlined and boxed, respectively, in bold. A single star shows p44/msp2 clusters with 99.2%–100% similarities and double stars show a cluster with 85.6% similarity. Two vertical bars and 6 arrows indicate Japanese p44/msp2 clusters and paralogs, respectively, which are distinct from the previously identified p44/msp2 (<73.1% similarity). A horizontal bar indicates percentage of sequence divergence. Accession numbers and location (Japan-Y [Yamanashi], Japan-S [Shizuoka], US [United States], and UK [United Kingdom]) are in parentheses.

The *p44*/*msp2* amplicons from 8 PCR-positive ticks and 1 PCR-positive mouse were cloned into a pCR2.1 vector with the TA Cloning Kit (Invitrogen, Carlsbad, CA, USA). Recombinant clones were randomly selected and 28 recombinant *p44*/*msp2* clones were sequenced with an ABI 3100-Avant Genetic Analyzer (Applied Biosystems, Foster City, CA, USA). A phylogenetic tree was constructed based on the alignment of Japanese *p44*/*msp2* sequences and the most closely related paralogs (220–400 bp) by using ClustalX (http://www-igbmc.u-strasbg.fr/BioInfo/ClustalX/), followed by the neighbor-joining method with 1,000 bootstrap resamplings (Figure 2). In this tree, the *p44*/*msp2* sequences obtained from *I. ovatus* were located mostly in clusters different from those where sequences from *I. persulcatus* were located, except for Tick41-1. This finding suggests that *A. phagocytophilum* in *I. ovatus* may encode *p44*/*msp2* paralogs distinct from those of *A. phagocytophilum* in *I. persulcatus*. A previous study suggested that the *p44*/*msp2* sequences from the United States and the United Kingdom can be divided into 27 similarity groups based on >90% similarities of DNA sequences, and most sequences from the United Kingdom are distinguishable from those from the United State because of the similarities <79% (15). Of 28 Japanese *p44*/*msp2* sequences in this study, 11 sequences with similarities >85.6% to the previously identified paralogs were probably divided into 8 similarity groups (Figure 2). Of the remaining 17 sequences with similarities <73.1%, 11 members that were grouped into 2 distinctive clusters (Figure 2) and 6 members that were individually located (Figure 2, arrows) were distinguishable from the 8 similarity groups. Thus, some *p44*/*msp2* paralogs of Japanese *A. phagocytphilum* are unique and distinct from those of *A. phgocytophlium* in other countries, although multiple copies of *p44* in the genome of an organism should be considered (*13*).

A partial sequence of the 16S rRNA gene of *A. phagocytophilum* (1.4 kb) from a *p44*/*msp2* PCR-positive mouse was amplified from spleen DNA with primers ER5-3, ER-R1, AP-F1, and AP-R1 (*12*), cloned, and sequenced. Similarities among 6 Japanese recombinant 16S rRNA sequences (GenBank accession nos. AY969010–AY969015) were 99.3%–99.6%. When compared with *A. phgocytophilum* human agent U02521, the similarities were 99.6%–99.8% between individual 16S rRNA cloned sequences and human agent U02521. Because we used pooled ticks to examine infection in mice, these sequence diversities may depend on genetic variants (or a heterogeneous population) of *A. phagocytophilum* from individual ticks. When the amplicon was directly sequenced, its sequence was identical with that of human agent U02521.

## Conclusions

We demonstrated that *A. phagocytophilum* infects *Ixodes* ticks in Japan, that both *I. persulcatus* and *I. ovatus* ticks are naturally infected with *A. phgocytophilum*, that *A. phagocytophilum* may be transmitted by *Ixodes* ticks because of organisms in the salivary glands of unfed and female adult ticks, and that immunocompromised mice can be infected with *A. phagocytophilum*. This study provides new information on the ecologic, biologic, and public health significance of *A. phagocytophilum* and emphasizes the threat of anaplasmosis in Japan.
